# Electrochemical Aptamer-Based Sensors for Rapid Point-of-Use Monitoring of the Mycotoxin Ochratoxin A Directly in a Food Stream

**DOI:** 10.3390/molecules23040912

**Published:** 2018-04-15

**Authors:** Jacob Somerson, Kevin W. Plaxco

**Affiliations:** 1 Interdepartmental program in Biomolecular Science and Engineering, University of California Santa Barbara, Santa Barbara, CA 93106, USA; jacob.somerson@lifesci.ucsb.edu; 2 Department of Chemistry and Biochemistry, University of California Santa Barbara, Santa Barbara, CA 93106, USA

**Keywords:** food taint, E-AB sensor, biosensor, mycotoxin, point-of-care, electrochemical sensor, aptamer

## Abstract

The ability to measure the concentration of specific small molecules continuously and in real-time in complex sample streams would impact many areas of agriculture, food safety, and food production. Monitoring for mycotoxin taint in real time during food processing, for example, could improve public health. Towards this end, we describe here an inexpensive electrochemical DNA-based sensor that supports real-time monitor of the mycotoxin ochratoxin A in a flowing stream of foodstuffs.

## 1. Introduction

Mycotoxins, a broad class of small molecule natural products produced by molds, are toxic to humans and other vertebrates. They primarily arise in foods stored in damp conditions and outbreaks are typically reported in the developing world. For example, after a jaundice outbreak in the eastern and central provinces of Kenya sickened 317 with 125 deaths a survey of maize products in the region found more than half were contaminated with mycotoxin [1]. The specific mycotoxin ochratoxin A contaminates a wide range of crops worldwide, including coffee beans, grapes, and many grains used both for human consumption and animal feed. It is primarily a nephrotoxin, but animal studies also show that it has liver toxicity, immune suppression, teratogenic, and carcinogenic activities [2]. Ochratoxin A contaminates a variety of crops worldwide with contaminating concentrations varying widely geographically and between crops [3]. Estimates of the tolerable human intake vary widely, as do regulatory limits of maximum acceptable concentration of ochratoxin A in human and animal foodstuffs [4]. The EU limits ochratoxin A concentrations on coffee beans to 5 µg/kg (roughly corresponding to 1 nM in brewed coffee) and ochratoxin A has been reported in concentrations as high as 1200 µg/kg (roughly corresponding to 200 nM in brewed coffee) [5,6]. 

The ability to rapidly detect or, better still, continuously monitor for mycotoxin contamination would significantly improve the safety of our food supply. The current state of the art in mycotoxin detection, however, depends on cumbersome laboratory-based techniques, with the most common being high-performance liquid chromatography [7]. While these techniques are sensitive and accurate, they are often inaccessible or economically challenging to deploy in the developing world [8]. An inexpensive detector to measure a mycotoxin at the point of use or the point of production would make measurement accessible to the developing world and improve the ease of measurement in the developing world. In response, we have developed an electrochemical aptamer-based biosensor (E-AB sensor) for the detection of this mycotoxin. 

**Figure 1:**
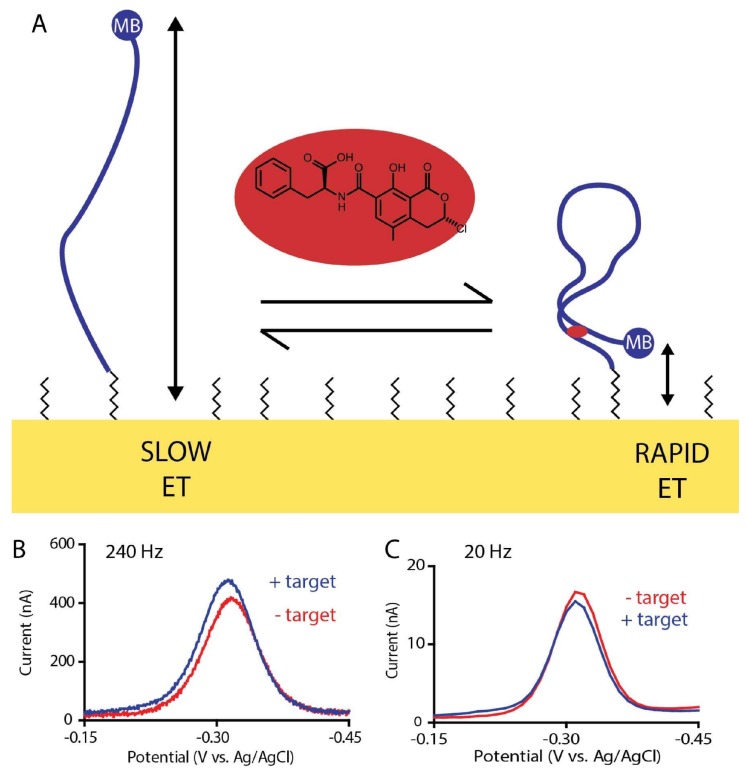
(**A**) The ochratoxin A-detecting E-AB sensor consists of an ochratoxin A-binding DNA aptamer modified attached via its 5′ end to a gold electrode using a six-carbon thiol linker (which forms part of a self-assembled monolayer) and modified on its 3′ end with a methylene blue redox reporter (blue circle, “MB”). The binding of ochratoxin A (red oval) alters the aptamer’s conformation, changing in turn the rate of electron transfer in a manner easily monitored using square wave voltammetry [10]. (**B**) At higher square wave frequencies square wave voltammetry is more sensitive to rapid electron transfer and, thus, target addition increases the observed current. (**C**) At lower frequencies, in contrast, target addition decreases the observed current.

The ochratoxin A-detecting E-AB sensors, like all sensors in this class, employ an electrode-bound, target-recognizing DNA aptamer modified with an electroactive “redox reporter” (Figure 1). Specific binding of the target alters the conformation of the aptamer, changing the efficiency of electron transfer between the reporter and the electrode. Interrogating this system using square wave voltammetry, an electrochemical measurement technique that is particularly sensitive to changes in transfer rates, we observe a change in measured current that is quantitatively related to the concentration of the target. Conveniently, such measurements are rapid, taking only seconds per measurement, and rely only on inexpensive equipment ranging from thousands of dollars for a research-grade instrument to under $100 for a simple, portable potentiostat [9]. Here we report an E-AB sensor for the detection of the mycotoxin ochratoxin A directly in flowing foodstuffs without the addition of any modifying reagents, allowing for the continuous monitoring of food processing workflows.

## 2. Results and Discussion

To create an ochratoxin A-detecting E-AB sensor we have employed an aptamer previously shown to bind this target with good specificity and affinity [11]. To date, several other groups have employed this aptamer for mycotoxin detection [11,12,13,14]. All of these previously-reported ochratoxin-detecting “aptasensors”, however, either require reagent additions or other processing steps, thus increasing the complexity and precluding continuous, real-time monitoring, or are based on fluorescence measurements, which are cumbersome and difficult to perform in foodstuffs due to autofluoresence and often significant light absorption and scattering. E-AB sensors, in contrast, are small (millimeter-scale), are supported by inexpensive, hand-held electronics [9] and provide a reagentless, single-step approach to measurement directly in highly complex media (e.g., whole blood, cell lysates, and foodstuffs) [15,16,17,18].

**Figure 2:**
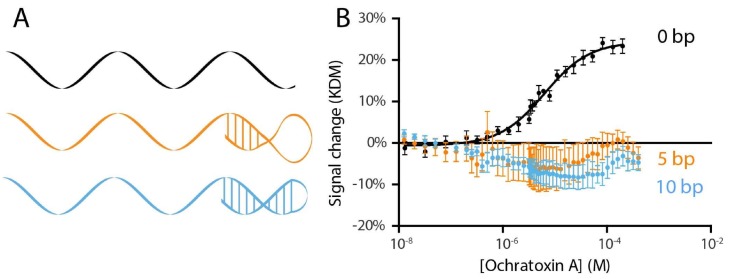
(**A**) To identify the E-AB sensor with the highest gain we explored the simplest linear aptamer sequence and two variants to which we added self-complementary tails. (**B**) Constructs with 5 and 10 self-complementary bases added to the 3′ end of the aptamer (orange and light blue) do not, however, exhibit any significant change in signal upon target addition. An aptamer lacking any self-complementary tail (black), in contrast, exhibits the expected binding curve. Error bars shown are standard error of the mean for four (no tail) or two (5 bp, 10 bp tail) independently-fabricated sensors.

**Figure 3:**
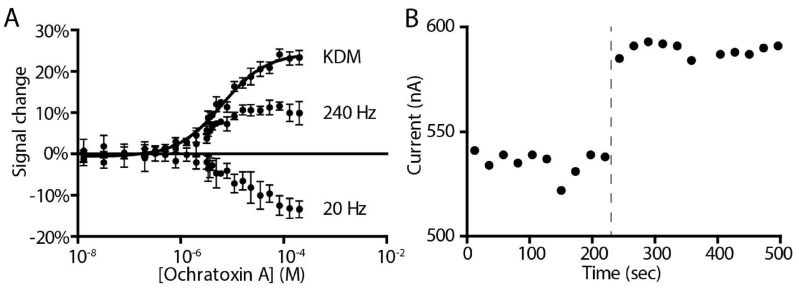
(**A**) The ochratoxin A-detecting sensor responds quantitatively to its target with a *K_D_* of 6.5 ± 0.6 µM in, as shown here, a simple buffer sample. Kinetic differential measurement (KDM), which takes the difference between measurements obtained at signal-on and signal-off square wave frequencies allows us to improve the signal magnitude and self-correction for a more stable signal [21]. Error bars shown for the 20 Hz and 240 Hz data are the standard deviation of four replicate electrodes. Error bars shown for KDM are the standard error of the mean for four replicate calculations. (**B**) The E-AB ochratoxin A sensor responds rapidly when challenged with the target. Shown is the current of a single sensor interrogated multiple times in succession in buffer both before and after the sudden addition of saturating (800 µM) target.

In order to support their use in E-AB sensors and many other sensing approaches an aptamer must undergo a conformational change upon target binding. Previously published works with this ochratoxin A aptamer have achieved this by adding to the sample solution a short DNA strand complementary to part of the aptamer sequence [11,12,13,14]. This creates an equilibrium between the partially double-stranded conformation (aptamer plus complement) and the free, properly folded aptamer. Since only the properly folded aptamer binds the target, target binding drives this equilibrium to the aptamer fold, coupling recognition with a large change in conformation [19,20]. The thermodynamics of this equilibrium (which is defined by the number of self-complementary bases in the complex and the stability of the aptamer fold) modulates the sensor’s signal gain (relative signal change upon target binding) and its affinity, with higher gain but poorer affinity being associated with stronger hybridization to the complement. Further limiting this approach, the complement is a separate piece of DNA that is “lost” during use and must be replenished by the user before the sensor can be re-used. To overcome this limitation, we instead coupled the complement to the aptamer sequence via an unstructured polythymine linker to create a single, self-complementary strand that cannot be washed away in a flowing sample stream, for example. Specifically, we created two constructs: one with the 10-base complement used in the earlier literature and a sensor with only five self-complementary bases (Figure 2A). Characterizing these constructs, however, we saw little E-AB signaling (Figure 2B). Ironically, however, we found that the parent aptamer already produces acceptable E-AB signal gain without the addition of any complementary sequence, presumably because it undergoes a binding-induced conformational change without requiring displacement of a complement. Specifically, we observe a 12% signal increase upon addition of saturating target to this sensor when it is interrogated at a square wave frequency of 240 Hz and, at “signal-off”, a 13% signal decrease when interrogated at 20 Hz (Figure 3).

The occurrence of both signal-on and signal-off behavior in the sensor provides an opportunity to employ kinetic differential measurement (KDM), a method of improving the gain and drift stability of E-AB sensors [21]. KDM does so by measuring two regimes of the electron transfer these sensors use as their signaling mechanism: a fast, “signal-on” regime where the target addition increases the electrochemical signal and a slow, “signal-off” regime where the target addition decreases the electrochemical signal (Figure 1). Taking the difference between these two measurements we improve the gain of the ochratoxin A-detecting sensor to 25%. The resultant sensor binds its target with a *K_D_* of 6.5 ± 0.6 µM when challenged in phosphate-buffered saline (Figure 3A). It is also rapid, fully responding to the addition of 800 µM target within the few seconds it takes to perform the requisite (two per time point) square wave voltammetric scans (Figure 3B).

**Figure 4:**
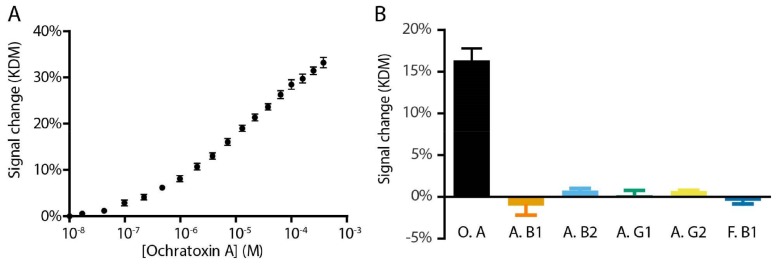
The E-AB ochratoxin sensor is selective enough to work when challenged directly in a realistically complex sample matrix, and highly specific for its target. (**A**) Shown here, for example, are data collected in flowing, undiluted coffee. Error bars shown are standard error of the mean for six replicate electrodes. (**B**) The E-sensor is also highly specific for its target, ochratoxin A (O. A); it does not respond to the mycotoxins, Aflatoxin B1 (A. B1), Aflatoxin B2 (A. B2), Aflatoxin G1 (A. G1), Aflatoxin G2 (A. G2), or Fumonisin B1 (F. B1). Each bar represents the KDM signal gain of sensors in buffer challenged with 10 µM of mycotoxin, error bars reflect the standard error of the mean for three replicate electrodes.

To achieve real utility as a point-of-care or point-of-production sensor a device must be selective enough to measure its target directly in the unfiltered, unadulterated media of interest, and be highly specific for that target. To illustrate the utility of our sensor for the measurement food taint in relevant foodstuffs we, thus, challenge it in unprocessed iced coffee and find that our sensor is selective enough to work directly in this undiluted, unadulterated commercial food product (Figure 4A). Under these conditions it exhibits a signal gain of 33% and a *K_D_* of 19 ± 2 µM. To demonstrate the value of our sensor in measuring only the target of interest, we also challenged it with five other mycotoxins and find that, as expected, our sensor responds only to the specific mycotoxin ochratoxin A (Figure 4B). 

The resulting E-AB sensor demonstrates a sensitive, specific, rapid single-step measurement of ochratoxin A concentrations directly within a relevant foodstuff. This enables point-of-care measurements in resource-limited areas, providing a fast, simple assurance against food taint.

## 3. Materials and Methods 

### 3.1. Materials

Ochratoxin A was purchased from Enzo Life Sciences (Farmingdale, NY, USA) and used as received without further purification. Ochratoxin A was dissolved in ethanol to a concentration of 61.9 µM (25 mg/mL) then further diluted for titration in the same media as measurements were performed. Aflatoxin B1, aflatoxin B2, aflatoxin G1, aflatoxin G2, and fumonisin B1 were purchased from Sigma Aldrich (St. Louis, MO, USA). 20X phosphate-buffered saline (PBS) was purchased from Sigma Aldrich (St. Louis, MO, USA), diluted to 1X concentration and pH corrected to 7.4 using sodium hydroxide and hydrochloric acid. Starbucks brand unsweetened iced coffee was purchased from Ralphs Supermarket (Santa Barbara, CA, USA) and used as received. Oligonucleotides were synthesized by Biosearch Technologies (Novato, CA, USA) and purified by dual HPLC. 6-mercapto-1-hexanol and tris(2-carboxyethyl)phosphine were purchased from Sigma Aldrich (St. Louis, MO, USA) and used as received.

### 3.2. Ochratoxin A Aptamer Sequences

Oligonucleotides were synthesized by Biosearch Technologies (Novato, CA, USA) and purified by dual HPLC. All constructs had a six-carbon thiol linker on the 5′ end and a methylene blue redox tag on the 3′ end. Self-complementary regions are shown underlined.

0 complementary bp

GATCG GGTGT GGGTG GCGTA AAGGG AGCAT CGGAC A

5 complementary bp

GATCG GGTGT GGGTG GCGTA AAGGG AGCAT CGGAC ATTTT TTGAT GC

10 complementary bp

GATCG GGTGT GGGTG GCGTA AAGGG AGCAT CGGAC ATTTT TTTGT CCGAT GC

### 3.3. Sensor Preparation

Our biosensor consists of a 2 mm diameter gold disk electrode (CH Instruments, Austin, TX, USA) coated with a mixed self-assembled monolayer of thiol-modified DNA strands and 6-mercapto-1-hexanol. Electrodes were physically polished and electrochemically cleaned as previously described [22]. DNA sequences were treated with a 1000-fold molar excess of tris(2-carboxyethyl)phosphine to reduce the 5′ disulfide modification to a free thiol. Following electrochemical cleaning, electrodes were immersed in 200 nM reduced DNA in 1X PBS at room temperature for 1 h and then moved to 20 mM 6-mercapto-1-hexanol solution in 1X PBS at 4 °C overnight.

### 3.4. Electrochemical Measurements

Electrochemical measurements were performed at room temperature using a CHI630C potentiostat with a CHI684 multiplexer (CH Instruments, Austin, TX, USA) and a standard-three electrode cell containing a platinum wire counter electrode and an Ag/AgCl reference electrode (both from CH Instruments, Austin, TX, USA). Square wave voltammetry was performed at 240 Hz using a potential step of 0.004 V and at 20 Hz using a potential step of 0.001 V, both using an amplitude of 0.025 V. All experiments were conducted in a closed-loop system with a continuous flow of test media (~1 mL/s) using a circulator pump (Cole-Parmer, Vernon Hills, IL, USA).
